# Serotonin Receptors and Their Involvement in Melanization of Sensory Cells in *Ciona intestinalis*

**DOI:** 10.3390/cells12081150

**Published:** 2023-04-13

**Authors:** Silvia Mercurio, Matteo Bozzo, Alessandro Pennati, Simona Candiani, Roberta Pennati

**Affiliations:** 1 Department of Environmental Science and Policy, Università degli Studi di Milano, 20133 Milan, Italy; sil.mercurio@gmail.com; 2 Dipartimento di Scienze della Terra, dell’Ambiente e della Vita, Università degli Studi di Genova, 16132 Genoa, Italy; matteo.bozzo@unige.it; 3 Institute of Zoology, University of Innsbruck, 6020 Innsbruck, Austria; alessandro.pennati@uibk.ac.at

**Keywords:** tunicate, ascidian, pigment, pigmented sensory organs, melanin synthesis, peripheral nervous system, epidermal sensory neurons, bipolar tail neurons, serotonin evolution, G-protein-coupled receptors

## Abstract

Serotonin (5-hydroxytryptamine (5-HT)) is a biogenic monoamine with pleiotropic functions. It exerts its roles by binding to specific 5-HT receptors (5HTRs) classified into different families and subtypes. Homologs of 5HTRs are widely present in invertebrates, but their expression and pharmacological characterization have been scarcely investigated. In particular, 5-HT has been localized in many tunicate species but only a few studies have investigated its physiological functions. Tunicates, including ascidians, are the sister group of vertebrates, and data about the role of 5-HTRs in these organisms are thus important for understanding 5-HT evolution among animals. In the present study, we identified and described 5HTRs in the ascidian *Ciona intestinalis*. During development, they showed broad expression patterns that appeared consistent with those reported in other species. Then, we investigated 5-HT roles in ascidian embryogenesis exposing *C. intestinalis* embryos to WAY-100635, an antagonist of the 5HT1A receptor, and explored the affected pathways in neural development and melanogenesis. Our results contribute to unraveling the multifaceted functions of 5-HT, revealing its involvement in sensory cell differentiation in ascidians.

## 1. Introduction

Serotonin (5-hydroxytryptamine (5-HT)) is a ubiquitous monoamine, mostly known to act as a neurotransmitter modulating many human physiological processes. Serotonergic neurons regulate mood, appetite, and sleep, as well as cognitive activities and social behavior [1,2]. In animals, 5-HT mediates several functions in the central and peripheral nervous systems too. Moreover, its involvement in chemotaxis and chemosignaling has been reported in unicellular organisms, and its diverse roles as an intracellular regulator of cell activity have been described in both vertebrates and invertebrates [2,3,4].

During vertebrate embryogenesis, 5-HT modulates neural migration, neurite outgrowth, as well as pigmentation and organogenesis, being involved in craniofacial development and orchestrating morphogenetic movements [5,6]. Its morphogenetic activity is also prominent in invertebrates and includes control of cleavage divisions, cell movement, and nervous system differentiation [7,8,9].

5-HT exerts its functions by binding to specific receptors that in humans have been classified into seven families (5HT1-7) and includes at least twenty subtypes [10]. 5HTR3 are ligand-gated ion channels while all the other serotonin receptors (5HTRs) are G-protein-coupled receptors that can stimulate or inhibit adenylyl cyclase upon bonding to the ligand. They are all expressed in the central nervous system (CNS) and various body districts, such as the gastrointestinal tract or cardiovascular system [10,11]. Homologs of 5HTRs are widely present in invertebrates from planarians [12] to mollusks [3,9] and insects [13,14]. Based on evolutionary studies, 5HTRs are among the oldest receptors of the rhodopsin-like family, as they appeared 700–750 million years ago, predating the invertebrate–vertebrate divergence estimated to have occurred about 600 million years ago [15,16,17]. The molecular and pharmacological characterization and the expression data of 5HTRs are available only for a few invertebrate species, mostly belonging to insect, mollusk, and nematode groups [17,18]. In *Drosophila melanogaster*, 5HTRs were found in the CNS of late embryos and adults where they control circadian rhythms and motor activities. On the contrary, during early embryogenesis, a 5HT_2Dro_ showing a pharmacological profile similar to human 5HT_2B_ was found to probably act as a sort of patterning gene [19,20,21]. In the mollusks *Lymnaea stagnalis* and *Aplysia californica*, 5HTRs were identified in CNS and peripheral structures, such as the heart, reproductive system, gills, and kidney [22,23,24]. Three 5HTR genes were also cloned in *Caenorhabditis elegans* and *Ascaris suum* but expression data were mostly absent [25,26,27]. In invertebrate deuterostomes, our current knowledge about 5HTRs is even more fragmentary and mainly related to echinoderms. In the genome of the sea urchin *Strongylocentrotus purpuratus*, four 5HTRs were found but their expression and function are still to be determined [28]. On the contrary, in *Hemicentrotus pulcherrimus*, a serotonin receptor, *5-HT-hpr*, similar to *A. californica 5-HT2*, has been identified and its expression has been well described during development [29]. In the sea cucumber *Apostichopus japonicus*, 5-*HT4R* was cloned and functionally studied in adults and its involvement in respiratory depression during animal aestivation was proposed [30]. Only a few genomic data are instead available for invertebrate chordates: in the amphioxus *Branchiostoma floridae* three 5HTRs were identified [31], while at least four 5HTRs have been found in the genome of the tunicate *Ciona intestinalis* [32].

Among tunicates, ascidians are the most studied organisms, and *Ciona intestinalis* and *Ciona robusta* are well-recognized model systems in developmental biology [33]. Their tadpole larva exhibits the typical chordate features, including a dorsal tubular CNS, made up of only about 330 cells arranged into the anterior sensory vesicle, the visceral ganglion, and the caudal nerve cord. The sensory vesicle houses two pigmented sensory organs: the otolith and the ocellus. The otolith is responsible for gravity perception and pigmentation is necessary for proper geotactic behavior of the larva [34]. The ocellus is a multicellular light-sensing organ formed by a cup-shaped pigmented cell, 3 lens cells, and almost 30 photoreceptor cells organized into 3 groups [35]. The peripheral nervous system is composed of epidermal sensory neurons scattered among the epidermis [36]. 

Serotonin has been localized in adults and larvae of several tunicate species [37,38], but only a few studies have investigated its physiological function. In the ascidian *Phallusia mammillata*, treatments at the gastrula stage with antagonists of the 5HT3, 5HT2B, and 5HT1A receptors induced typical defects including nervous system anomalies [39,40]. Interestingly, treatment with WAY-100635, a potent and selective 5HT1A receptor antagonist [41], caused defective pigmentation in the pigmented sensory organs [40], suggesting a conserved role of 5-HT in melanogenesis between ascidians and vertebrates [42,43]. Any attempts to immunolocalize 5-HT failed in *C. intestinalis* samples, but the expression of the *tryptophan hydroxylase* (*TPH*), the rate-limiting enzyme in the biosynthesis of 5-HT, has been characterized during embryo development. At the larva stage, *Ci-TPH* transcripts were detected in a few cells of the CNS organized into two clusters at the level of the visceral ganglion; moreover, in the tail, they were present at the level of the neuro–muscular junctions [44]. A *5-HT transporter-like* (*SERT*) gene was also detected and its expression was investigated. *Ci-SERT* appeared to be expressed in peculiar dopaminergic cells, called coronet cells, located in the sensory vesicle and pharmacological studies demonstrated that its function was related to spontaneous and light-triggered swimming [45]. Although the serotonergic system has been deeply investigated in *C. intestinalis*, the distribution and role of the 5-HT receptors have not been explored yet.

To provide a comprehensive picture of ascidian 5HTRs, in this work, we searched and identified 5HTRs in the *C. intestinalis* genome, and then we described their expression patterns during development. We further investigated 5-HT roles in ascidian embryogenesis exposing *C. intestinalis* embryos to WAY-100635 and exploring the developmental pathways affected by this 5HT1A antagonist in neural development and melanogenesis.

## 2. Materials and Methods

### 2.1. Animal Maintenance

Specimens of *Ciona intestinalis* were collected by the fishing service of the station Biologique de Roscoff (France). Animals were kept in aquaria as previously described [46,47]. All the experiments were performed at 18 ± 1 °C. For each experiment, gametes were obtained surgically from three adults and in vitro cross-fertilization was performed. Embryos were reared until the stage of interest, fixed in 4% paraformaldehyde, 0.5 M NaCl, and 0.1 M 3-(N-morpholino)-propanesulfonic acid (pH 7.5; MOPS fixative), and then stored at −20 °C [48].

### 2.2. RNA Preparation

Total RNA was isolated from a pool of mixed *C. intestinalis* embryos using Trizol reagent (Invitrogen, San Diego, CA, USA) and then treated with RNase-free DNase I (Ambion Europe Ltd., Warrington, UK) to remove genomic DNA. The first strand cDNA synthesis reaction from total RNA was catalyzed by Superscript III reverse transcriptase using oligo(dT) primers (Invitrogen, San Diego, CA, USA).

### 2.3. Identification of 5-HT Receptor Sequences and Molecular Cloning

*Ciona* 5-HT receptor sequences were identified by BLAST search on the NCBI (http://www.ncbi.nlm.nih.gov, last accessed on 27 February 2023) and ANISEED (http://www.aniseed.cnrs.fr/, last accessed on 27 February 2023) databases by using as queries different vertebrate sequences encoding for each class of receptors and PCR-amplified from cDNA of *C. intestinalis* embryos (sequences and primers are listed in the Supplementary Materials, Additional File S1, Tables S1 and S2). The amplicons were sequence verified (Eurofins Genomics, Vimodrone, Milano, Italy) and cloned into a pcRII-TOPO-TA vector (Invitrogen, Carlsbad, CA, USA). The isolated cDNA clones were also used as a template for antisense and sense riboprobes for the *in situ* hybridization experiments.

### 2.4. Phylogenetic Analysis of 5-HT Receptors (5HTRs)

Phylogenetic analysis was performed using peptide sequences of 5-HT receptors retrieved from the NCBI and Aniseed databases excluding uninformative proteins. The protein set was aligned by Clustal W with default parameters in MEGA 11 [49]. The gapped regions corresponding to poorly conserved N-terminal and C-terminal domains and intracellular and extracellular loops were removed (Supplementary Materials, Additional File S2). A phylogenetic tree was inferred from this dataset using the maximum likelihood estimation (ML) in MEGA11 with the LG + G. The rate variation among sites was modeled with a gamma distribution (5 categories) with shape parameter = 1.0510 in an analysis involving 47 amino acid sequences. There was a total of 237 positions in the final dataset. For the NJ method, evolutionary distances were computed using the p-distance method and are expressed as numbers of amino acid substitutions per site. Bootstrap confidence limits were obtained by 500 replicates in both ML and NJ analysis. Tree files were visualized with MEGA 11. Sequences used in the phylogenetic analysis are found in the Supplementary Materials, Additional File S1, Table S3. The tree was rooted using rat metabotropic glutamate receptor sequences as an outgroup (as previously reported [50]).

### 2.5. WAY-100635 Exposure during Embryogenesis

WAY-100635 (MW = 538.64) was purchased from Merck. A stock solution of 10 mM was made in distilled water and the final test concentrations (1 µM, 10 µM, 25 µM, and 50 µM) were prepared by dilution in artificial seawater buffered with 1 MHEPES (pH 8; ASWH). Concentrations were defined based on previously published research [39,40] and preliminary tests. Micromolar concentrations were necessary as whole embryos with all their envelopes were exposed to the test solutions. New solutions were made every time. For each experiment, about 100 embryos at the two-cell stage were moved to Petri dishes filled with 10 mL of the test solutions and cultured until the stage of interest. Experiments were performed in 3 replicates and considered reliable only if ≥80% of controls developed normally. Samples were then fixed in MOPS fixative for 90 min, dehydrated, and stored at −20 °C.

### 2.6. Whole Mount in Situ Hybridization

To describe gene expression profiles during embryogenesis and investigate WAY100635 effects, a protocol for whole mount in situ hybridization (WISH) was employed as previously described [51]. Briefly, after rehydration, embryos and larvae were permeabilized with proteinase K, post-fixed in MOPS fixative, and pre-hybridized in 50% formamide, 5 × SSC, 100 μg/mL yeast RNA, 50 μg/mL heparin, and 0.1% Tween-20 at 50 °C for 2 h. Hybridization was performed overnight. After several washes in 50% formamide, 5 × SSC, 0.1% Tween-20, and phosphate-buffered saline (PBS) with 0.1% Tween-20 (PBT), the embryos were moved into a blocking solution (25% goat serum and 75% PBT) for 2 h. Then, the samples were incubated overnight at 4 °C in the blocking solution with anti-Digoxigenin-AP antibody (Merck, 1:2000). Embryos were rinsed in PBT, and the hybridization signal was obtained in alkaline phosphatase-labeled buffer (100 mM NaCl, 100 mM Tris HCl, pH 9.5, 50 mM MgCl_2_, and 0.1% Tween-20) + 4-Nitrotetrazolium Blue chloride and l 5-Bromo-4-chloro-3-indolyl phosphate p-toluidine salt (Merck). When a proper stain was detected, embryos were fixed, mounted in 80% glycerol in PBS, and observed under an optical microscope equipped with a Leica DFC-320 Camera.

Digoxigenin-labelled riboprobes were synthesized as reported in [51]. Clones used for in situ hybridization were amplified from *C. intestinalis* cDNA or obtained from the *Ciona* Gene Collection release 1 [52]; the full list of probes and primers is provided in Table S2.

Labeled embryos were counterstained with 1% Ponceau S in 1% acetic acid, embedded in resin, and sectioned at 3 μm.

## 3. Results

### 3.1. Phylogenetic Analysis of 5-HT Receptors (5HTRs)

By BLAST search, we identified five serotonin receptors in the *Ciona* genome. PCR assays allowed us to isolate all identified sequences from embryonic cDNA of *C. intestinalis*, except for the *5HT2* orthologs.

**Figure 1:**
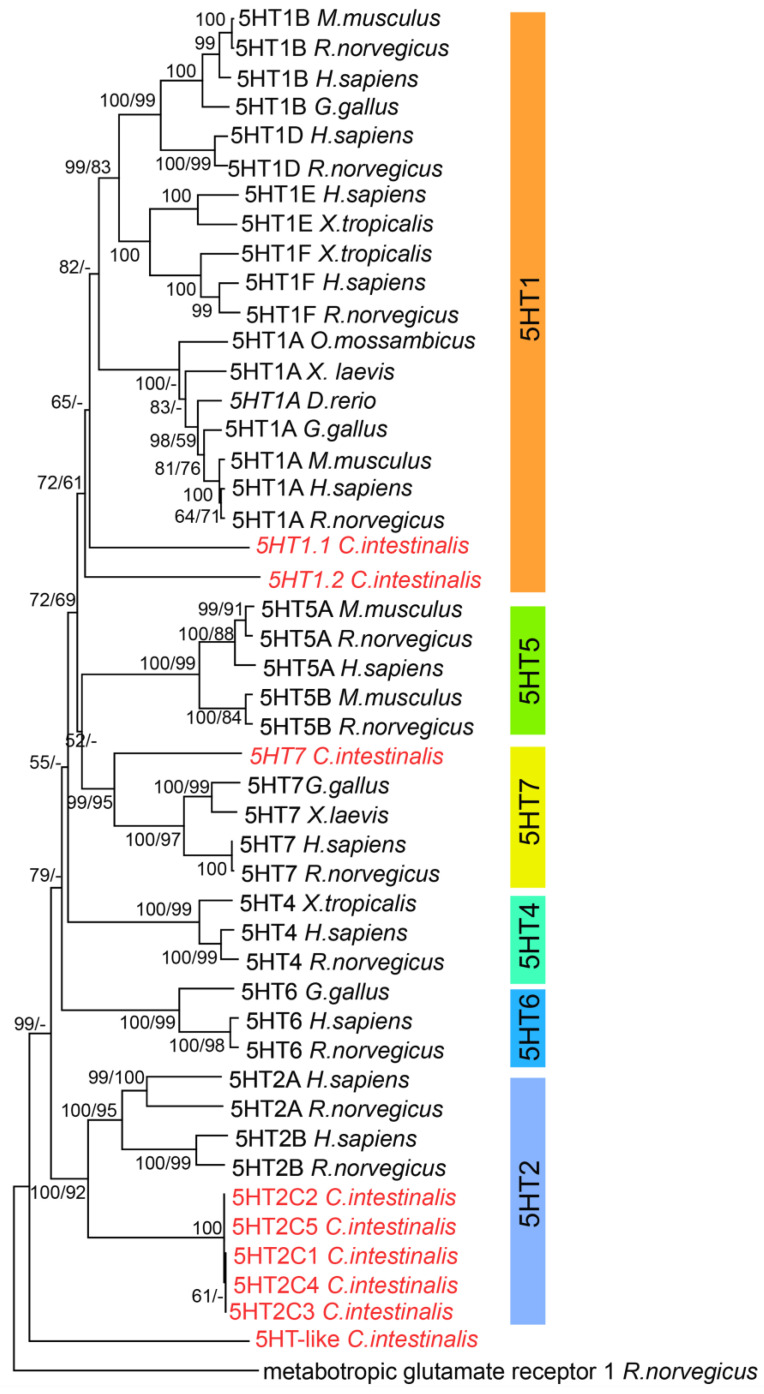
Phylogenetic tree of metabotropic serotonin receptors in vertebrates and *Ciona*. The evolutionary history was inferred using both the maximum likelihood (ML) and neighbor-joining (NJ) methods. The NJ tree is shown with bootstrap values for both NJ and ML analyses (first and second values, respectively). The percentage of replicate trees in which the associated taxa clustered together in the bootstrap test (500 replicates) is shown next to the branches. Bootstrap identical values in both NJ and MK are shown only once and values under 50% were collapsed. Differences in the two analyses are reported in the ML with an absent value. The tree was rooted using rat metabotropic glutamate receptor sequences as an outgroup.

Then, we constructed a phylogenetic tree to investigate the evolutionary relationships between the serotonin receptors of *Ciona* and the serotonin receptors of vertebrates. The neighbor-joining phylogenetic tree (Figure 1) showed that *Ciona* possesses two sequences, named 5HT1.1 and 5HT1.2, that are basal to all 5HT1 paralogs of vertebrates, a 5HT2 ortholog (with five protein isoforms) and a 5HT7 ortholog, and a highly divergent sequence that we named 5HT-like, which seems to be basal to all G-coupled 5HT classes. This phylogeny was also mostly supported by our ML phylogenetic reconstruction, with a few exceptions. The maximum likelihood tree placed the *Ciona* 5HT1.1 and 5HT1.2 within the 5HT1 clade, failing to resolve their homology to a particular subtype of 5HT1. Similarly, 5HT-like was inserted into the clade of 5HT1/2/4/5/6 receptors but without resolving its position.

### 3.2. Expression Profile of 5HTRs

**Figure 2:**
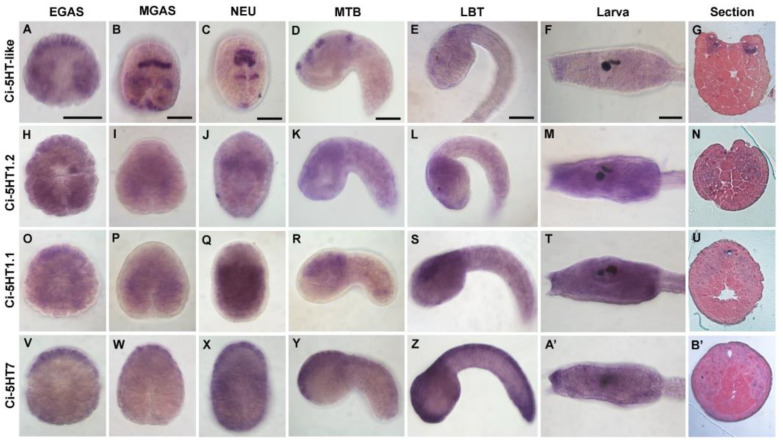
Expression profile of serotonin receptors during *C. intestinalis* development. Expression of 5HTRs by whole mount *in situ* hybridization in five developmental stages: early gastrula stage (EGAS) (**A**,**H**,**O**,**V**); mid gastrula stage (MGAS) (**B**,**I**,**P**,**W**); neurula stage (NEU) (**C**,**J**,**Q**,**X**); mid-tailbud stage (MTB) (**D**,**K**,**R**,**Y**); late tailbud stage (LTB) (**E**,**L**,**S**,**Z**); and larva stage (only trunk and the beginning of the tail is shown; **F**,**M**,**T**,**A’**). Representative microscopy transverse sections of hybridized samples are shown in **G** (neurula stage), **N** (initial tailbud stage), and **U** and **B’** (mid-tailbud stage). Scale bars: 25 µm (**B**–**E**); 50 µm (**A**,**F**).

In Figure 2, the expression patterns of four out of the five identified 5HTRs are shown in five representative developmental stages. *5HT-like* displayed a dynamic expression pattern during development, mainly with strong signals at the single-cell level (Figure 2A–G). At the early gastrula stage (stage 11; [53]), its transcripts were detected in a row of six a-line-derived cells and faintly in two groups of B-line-derived cells symmetrically distributed around the blastopore (Figure 2A). At stage 12 (6-row stage; [53]), the signal was still present in the a-line lineage, comprising cells of the III row of the neural plate, but it also extended to the A-line-derived 9.29 pair cells of the I row and to the posterior b-line-derived cells from which the bipolar tail neurons (BTN) of the larva are derived (Figure 2B). At the neurula stage (stage 15; [53]), a hybridization signal was observed in 3 rows of cells of the neural plate, i.e., precursors of the anterior sensory vesicle and pigment cells, in A-line-derived cells that contributed to the formation of the visceral ganglion and in posterior progenitors of BTNs (Figure 2C). At the mid-tailbud stage (stage 20/21, [53]), the transcripts were restricted to a few cells of the developing palps, anterior sensory vesicles including progenitors of the pigmented organs, and visceral ganglion (Figure 2D). From the late tailbud stage, the expression of 5HT-like was not clearly detectable, as only a faint signal was observed in larva palps (Figure 2E,F).

The other *5HTR* genes showed a much broader expression. *5HT1.1* and *5HT1.2* genes, both clustering with the *5HT1* receptors of other species (Figure 1), showed a similar expression pattern, overlapping in most developmental stages (Figure 2H–U). At the early gastrula stage, a faint hybridization signal was first detected in most of the cells with a stronger signal in anterior a-line-derived cells (Figure 2H,O). Then, the signal extended into the mesenchyme territories (Figure 2I,P), and at the neurula stage both genes showed a sharp limit of expression at the level of anterior mesodermal masses (Figure 2J,Q). At this stage, the *5HT1.1* signal was stronger and spread further in the posterior part of the embryos. At the mid-tailbud stage, *5HT1.2* and *5HT1.1* expression was localized in trunk mesoderm cells and in the sensory vesicle (Figure 2K,R). This expression persisted until the late-tailbud stage (Figure 2L,S), but at the larva stage, the hybridization signal appeared widely distributed all over the trunk epidermis while no stain was observed in the tail (Figure 2M,T).

*5HT7* gene expression (Figure 2V–B’) was detected at the early gastrula stage in a-line-derived cells forming the anterior-most row of six cells. Then, at stage 12, the hybridization signal persisted in a row of anterior cells (Figure 2W) that were also detectable at the neurula stage (Figure 2X). At the mid-tailbud stage, transcripts extended backward to include the dorsal midline ectoderm and ventrally towards palp progenitors (Figure 2Y). Late tailbud embryos showed an intense hybridization signal along the dorsal midline, from the palp territory to the tip of the tail (Figure 2Z). At the larva stage, *5HT7* expression appeared faintly distributed in both trunk and tail (Figure 2A’).

### 3.3. WAY-100635 Exposure Decreased Melanin Content in Ascidian Pigmented Sensory Organs

**Figure 3:**
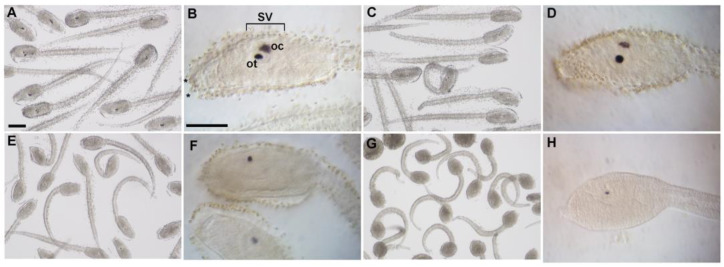
Effects of WAY-100635 treatment on larval morphology. Control samples (**A**,**B**) and larvae exposed to 10 µM (**C**,**D**), 25 µM (**E**,**F**), and 50 µM (**G**,**H**) WAY-100635. General morphology and magnification of larval trunk are displayed. Scale bars: 100 µm (**A**) and 50 µm (**B**). Abbreviations: SV = sensory vesicle; ot = otolith; oc = ocellus; * = palps.

Control samples displayed a normal morphology with an elongated trunk and a straight motile tail (Figure 3A). Larvae exposed to increasing concentrations of WAY-100635 showed altered phenotypes from 25 µM: the trunk appeared roundish with malformed palps and the tail was often curved (Figure 3E,F). In the sensory vesicle of control samples, the two pigmented organs, the otolith and the ocellus, were well developed (100%, N = 71; Figure 3B); and from 10 µM WAY-100635, a reduction in pigment was instead observed in treated samples (Figure 3C–H): in 79% of the swimming larvae (N = 75), the pigment organs were recognizable by their shape but melanin content was drastically reduced. The ocellus seemed to be more affected by the treatment as it was completely demelanized from 25 µM WAY-100635 (96.6% of hatched larvae, N = 59, Figure 3F), whereas some granules of melanin were still observable in the otolith of all the larvae treated with 50 µM WAY-100635 (100% of the hatched larvae, N = 59, Figure 3H).

### 3.4. 5-HT Is Involved in Pigment Cell Terminal Differentiation

**Figure 4:**
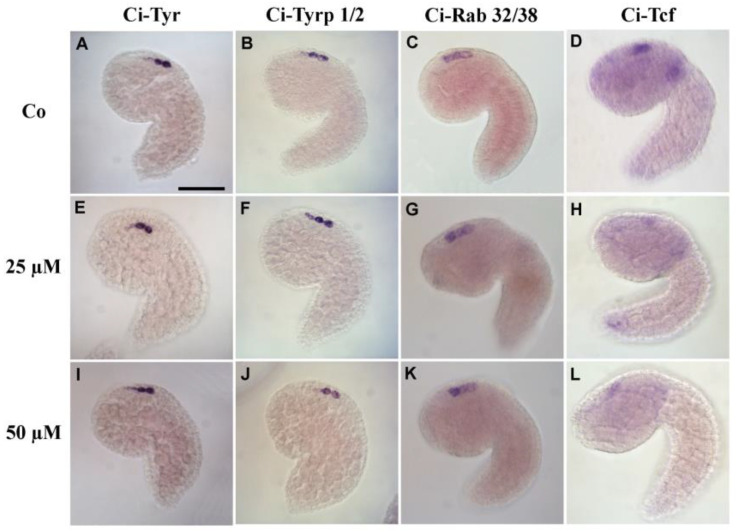
Effects of WAY-100635 treatment on genes involved in pigment cell formation. Expression patterns of *Ci-Tyr*, *Ci-Trp 1/2*, *Ci-Rab 32/38,* and *Ci-Tcf* in control (Co) (**A**–**D**) and embryos exposed to 25 (**E**–**H**) and 50 (**I**–**L**) µM WAY-100635 at mid-tailbud stage. Scale bar: 50 µm.

To test whether the depigmentation of the sensory organs was related to alterations of the melanin biosynthetic pathway, we analyzed the expression of *C. intestinalis* tyrosinase (*Ci-Tyr*) and tyrosinase-related-protein 1/2 (*Ci-Tyrp 1/2*) by whole mount in situ hybridization. At the mid-tailbud stage, these genes are expressed in pigment cell precursors, a11.193/a11.194 and a11.195/a11.196 pairs, with a stronger signal in the posterior ones [54,55,56]. Comparing controls and embryos exposed to WAY-100635, we did not observe differences in gene expression (Figure 4A,B,E,F,I,J).

*Ci-Rab 32/38* plays a key role in melanosome biogenesis and its dominant-negative mutation resulted in hypopigmentation of *C. intestinalis* pigmented sensory organs [57,58]. At the tailbud stage, *Ci-Rab 32/38* transcripts were detected in pigment cell precursors in both controls and embryos treated with WAY-100635, and no difference was noticed between the experimental groups (Figure 4C,G,K).

*Ci-Tcf* is the only gene known to be specifically expressed in the precursor cells of the ocellus and otolith, i.e., in the a11.193 pairs at mid-tailbud stage [56,59] as well as in mesodermal cells [60]. Perturbation of *Ci-Tcf* expression led to larval sensory organs being only partially melanized, suggesting a role in pigment cell terminal differentiation [59]. Performing in situ hybridization with *Ci-Tcf* probe, we found that 52.6% of 25 µM WAY-100635 and 71% of 50 µM WAY-100635 displayed a drastic decrease in its signal compared with controls (Figure 4D,H,L).

### 3.5. Pharmacological Inactivation of 5HTRs Disrupts the Development of Epidermal Sensory Neurons

**Figure 5:**
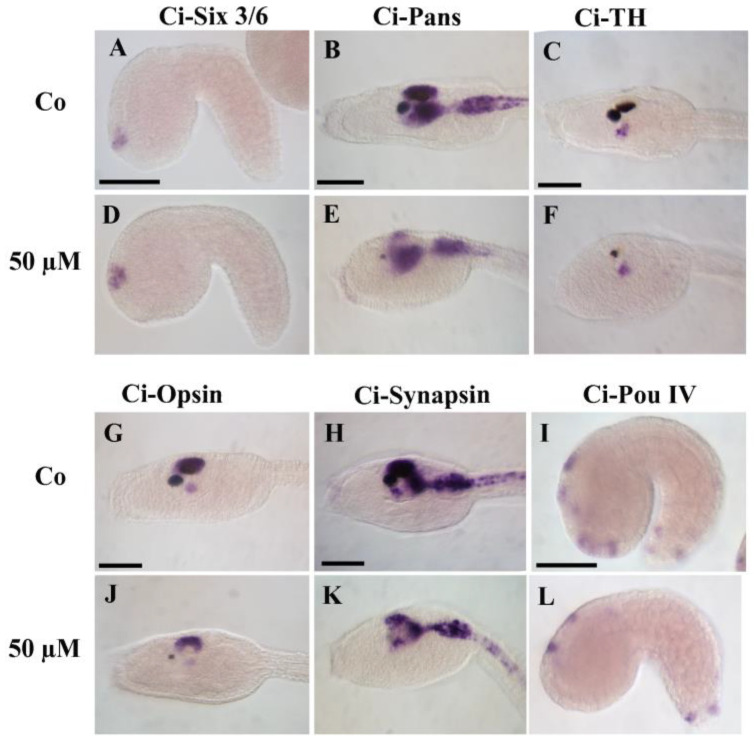
Effects of WAY-100635 on neural differentiation. Expression pattern of neural markers in mid-tailbud embryos (*Ci-Six 3/6*: (**A**,**D**); *Ci-Pou IV*: (**I**,**L**)) and larvae (*Ci-Pans*: (**B**,**E**); *Ci-TH*: (**C**,**F**); *Ci-Opsin*: (**G**,**J**); and *Ci-Synapsin*: (**H**,**K**)) of *C. intestinalis*. Scale bars: 50 µm.

The effects of WAY-100635 treatment on nervous system development were characterized by in situ hybridization with neural-specific markers (Figure 5).

*Ci-Six3/6* is expressed in cells that contribute to the anterior-most regions of the sensory vesicle [61]; at mid-tailbud stage, it is present in territory adjacent to the pigment cell precursors [57]. *Ci-Pans* is considered a pan-neural gene, expressed in most of the central nervous system of *C. intestinalis* larvae [62], including posterior sensory vesicles, visceral ganglion, and caudal neural tube. The expression of these genes did not appear to be affected by WAY-100635 treatments, as the hybridization signal was comparable between controls and treated samples (Figure 5A,B,D,E). To test if treatment affected neural differentiation, we marked dopaminergic neurons with a *Ci-TH* probe [63] and photoreceptors with a *Ci-Opsin1* probe [64]. In both cases, no differences in gene expression were observed between controls and samples exposed to 50 µM WAY-100635 (Figure 5C,F,G,J). As the role of serotonin in neurite outgrowth is well documented [65,66], we investigated the effects of the pharmacological treatments on *Ci-Synapsin*, a gene specifically expressed in the neural circuits of post-mitotic neurons [67]. Our results showed that WAY-100635 did not affect the pattern of this gene (Figure 5H,K). Finally, we checked the development of the peripheral nervous system by analyzing the expression of *Ci-Pou IV*, a specific marker of sensory neurons [68]. At mid-tailbud stage, the hybridization signal is present in the developing epidermal sensory neurons. Particularly, in the trunk *Ci-Pou IV* marks three pairs of epidermal cells and two pairs of dorsal ectodermal cells; in the tail, transcripts are present in both ventral and dorsal epidermal sensory neurons as well as the bipolar tail neurons. Comparing controls and samples exposed to 50 µM WAY-100635, we found that deactivation of 5HTR disrupted the development of both palps and caudal epidermal neurons with the only exception of two neurons at the tip of the tail. *Ci-Pou IV* remained expressed only in precursors of trunk epidermal sensory neurons (Figure 5I,L).

## 4. Discussion

Serotonin (5-HT) is a biogenic monoamine that activates one of the largest families of G-protein-coupled receptors [16]. In invertebrates, this neurotransmitter is involved in numerous physiological processes, and diverse receptor subtypes have been identified as well [18]. However, our current understanding of 5HTR distribution and roles among invertebrates is still fragmentary and their functional and pharmacological characterization is limited to a few phyla. 

In the genome of *C. intestinalis* we identified five *5HTRs*: *Ci-5HT1.1* and *Ci-5HT1.2*, which appeared basal to all *5HT1* paralogs of vertebrates, *Ci-5HT2*, *Ci-5HT7*, and a highly divergent gene, *Ci-5HT-like*, which appeared basal to all G-coupled *5HT* classes. The Aniseed database (https://www.aniseed.fr; last accessed on: 27 March 2023) reported expression levels of each gene obtained by RNA sequencing experiments in different developmental stages. For all *5HTRs*, the level of expressions was very low, ranging from 0.092 to 7 FPKM (fragments per kilobase million), with the only exception of *5HT-like* which was found at very high levels, up to 106 FPKM (Supplementary Materials, Additional File S1, Table S4 and Figure S1). During *C. intestinalis* embryogenesis, transcripts of only four *5HTRs* out of the five identified genes were successfully detected by in situ hybridization, and their expression pattern was described from the gastrula to larva stage. Despite the efforts, all the attempts to clone *Ci-5HT2* failed even if different amplification conditions and cDNA libraries were employed. Further analysis of the annotated sequence could help to solve the characterization of this gene. All *5HTRs* were expressed in different embryonic districts, confirming the wide distribution of serotonin receptors already reported in many animals [6,10,17]. In vertebrates, *5HTRs* were found in different areas of the central nervous system (CNS), in the vascular system, gastrointestinal tracts, as well as in muscles and epidermis [2,5,6,10,43,65,66,69]. Among invertebrates, most of the studies were performed on mollusks and arthropods. In *Aplysia californica*, three *5HTRs* were cloned: *Ap5-HTB1* was identified in the reproductive system, *Ap5-HT2B* was nervous system-specific and *5HT1Ap* was found in different structures such as gills, heart, CNS, kidney, and ovotestis [17,18,22]. Similarly, in the pond snail *Lymnaea stagnalis*, *5HTLym* was detected in the CNS and adult heart, while *5HT2Lym* was mainly present in the esophagus, salivary gland, sperm-oviduct, and heart [17,18,23,24]. In *Drosophila melanogaster*, expression of *5HT1B* receptor started in late embryonic stages in CNS, and persisted in the same territories in adults [70]. On the contrary, *5HT_2Dro_* was expressed both in the adult stage and during embryogenesis and was involved in segmentation processes [19]. In the armyworm *Mythimna separate*, two isoforms of the *5HT7* receptor were characterized and they were co-expressed in all the analyzed tissues, including the brain, antennae, wings, and gut [14]. The wide expression patterns observed in embryos and larvae of *C. intestinalis* were thus consistent with those reported in many other species. Noteworthy, even if sometimes the hybridization signal was faint, transcripts of all the *5HTRs* were detected in CNS lineage, suggesting a role in neural development as reported in vertebrates [6,69].

To get insight into serotonin functions during ascidian development, we exposed *C. intestinalis* embryos to WAY-100635, a potent and selective antagonist of the 5HT1A receptor [41]. Mammalian and invertebrate 5-HT receptors often showed different pharmacological properties, and these differences were ascribed to both different methodological approaches and structural diversity among receptors. However, WAY-100635 has been commonly tested in many invertebrate species, including ascidians [18,40,44]. Pharmacological characterization of 5HTRs was performed mainly in arthropods and this drug was demonstrated to act as an antagonist, or in a few cases as an inverse agonist, of 5HT1-like receptors [18]; however, interaction with other receptor subtypes has also been demonstrated [14].

As reported in the ascidian *Phallusia mammillata* [40], WAY-100635 induced malformations at the anterior-most structures also in *C. intestinalis* larvae: at high concentrations the trunk was roundish and, although present, the papillae appeared fused at the base. In addition, the two pigmented sensory organs inside the sensory vesicle showed a drastic reduction in melanin content. Melanin synthesis is a complex biochemical process that requires the tyrosinase enzyme (Tyr) and two tyrosinase-related proteins, Tyrp-1 and Tyrp-2, which are structurally related to tyrosinase [71]. In *C. intestinalis*, there is a single gene encoding for *Ci-Tyr* and two genes, *Ci-Tryp 1/2-a* and *Ci-Tyrp 1/2-b*, are defined as the ancestral forms of the two *Tyrp* of vertebrates [72]. These three genes show the same expression pattern starting from the gastrula stage and are considered specific markers of pigment cell lineage [54]. It was demonstrated that 5-HT is involved in vertebrate melanogenesis and the pathway has been investigated in different model organisms and human cell cultures. For example, 5-HT induced melanin synthesis in different cell lines and zebrafish embryos via 5HT2A receptor by upregulating *Tyr* and its related enzymes [42,73,74]. Fluoxetine, a selective 5-HT reuptake inhibitor, stimulated melanin production in human melanocytes and in zebrafish through 5HT1A/2A and its effect was inhibited by WAY-100635 [42]. Similar results were obtained in mice: 5-HT induced melanogenesis mainly through the upregulation of *Tryp* expression and treatment with antagonists of 5HT1A/1B and 5HT7 receptors could partially block the effect [43]. These results clearly suggested that 5-HT interaction with its receptors is required for proper melanogenesis in vertebrates. To test this hypothesis in *C. intestinalis*, we investigated the expression of *Ci-Tyr* and *Ci-Tyrp 1/2* genes in embryos exposed to increasing doses of WAY-100635, but no difference was observed between the control and treated samples. Similarly, the expression of *Ci-Rab 32/38*, a gene involved in melanosome formation [58], was not altered by drug exposure, suggesting that the main gene network controlling melanogenesis in *C. intestinalis* was not directly affected by WAY-100635. In this species, the only marker known to be specific to the precursors of the two pigmented sensory organs, the ocellus and the otolith, is *Ci-Tcf*, a downstream effector of the canonical Wnt signaling pathway [75]. For proper pigment cell development, FGF-Wnt crosstalk is strictly necessary: the FGF signaling cascade makes pigment cell precursors competent to respond to the Wnt pathway by directly controlling *Ci-Tcf* transcription through *Ci-Ets1/2* [57]. In vertebrates, different studies have suggested 5-HT interaction with the Wnt pathway: although the molecular mechanism was not always clarified, the interaction between glycogen synthase kinase-3 (GSK3) and 5HTRs type 1 and 2 has been demonstrated [76,77,78]. GSK3 is a ubiquitous protein kinase and the isoform GSK-3β plays a key role in the canonical Wnt pathway, being a modulator of β-catenin translocation to the nucleus. WAY-100635 treatment drastically reduced *Ci-Tcf* expression in *C. intestinalis* embryos, suggesting that the interaction between 5-HT and Wnt signaling could be conserved in ascidians. The role of *Ci-Tcf* in pigment cell differentiation has been already reported, and perturbation of *Ci-Tcf* resulted in defective pigment cell melanization [59]. WAY-100635 inactivation of GSK-3β, preventing nuclear β-catenin, has been demonstrated in neural precursors cells [77], providing a possible explanation of the observed down-regulation of *Ci-Tcf* expression induced by WAY-100635 in *C. intestinalis*. A similar mechanism could be involved in the effects observed in the peripheral nervous system (PNS). Our results showed that WAY-100635 treatment did not affect larva CNS development: the general architecture as well as differentiation of neural populations were normal in control and treated embryos while caudal epidermal neurons (i.e., PNS) failed to differentiate. In the *C. intestinalis* tail, the PNS consists of epidermal sensory neurons (ESN) distributed along the epidermis. During embryogenesis, these mechanoreceptors arise from the midline epidermis, where expression of *Ci-5HT7* was also observed, and they are selectively induced by FGF ligands. The gene regulatory network specifying ESN displays a high degree of complexity and comprised numerous transcription factors, such as Msxb and Nkx-C as upstream elements, or Pou IV and miR-124 as final nodes. This gene circuit is differentially initiated in the dorsal and ventral midlines but common posterior signals control the maintenance and the differentiation of this network [79,80], in which 5HTRs are likely to be involved. Indeed, several pathways contribute to ESN differentiation, including retinoic acid (RA), FGF/MAPK, and canonical Wnt pathways. In particular, the tail comprises distinct epidermal territories with different responsiveness to RA (in the anterior tail), FGF/MAPK signal, and the Wnt pathway (towards the tail tip), and the antagonism between them controls the anterior–posterior patterning of the caudal epidermal neurons [81,82]. In this context, the lack of most of the caudal sensory neurons detected in WAY-100635-treated embryos could be related to drug interference, with these complex interactions between diffusible molecules leading to disruption of key developmental processes. Interference with FGF signaling could also explain the observed malformations in the nervous system. Indeed, it has been demonstrated that the FGF-MAPK pathway is required for anterior neural plate patterning, from which palps and pigment cells arise [57,83], and for specification of BTNs which form lateral to the neural plate and then migrate in the posterior caudal region [84]. According to our results, the precursors of all these structures expressed at least one type of 5HTRs during *C. intestinalis* development. *Ci-5HT-like* was specifically observed in progenitors of the anterior sensory vesicle including the pigmented organs, some of the papillary neurons, and the posterior precursors of the BTNs. *Ci-5HT1.1* and *Ci-5HT1.2* were expressed in the developing sensory vesicles and *Ci-5HT7* transcripts were observed along the dorsal midline, from the palp territory to the tip of the tail. Anyway, WAY-100635 interactions with other receptors cannot be completely excluded. Indeed, in mammals, this compound has also shown an affinity for dopamine and adrenergic receptors [85,86]. In the *Ciona* genome, no dopamine-like (either D1 or D2 types) receptor was found, while four adrenergic-like receptors (ADRs, two *ADRβ-like* and two *ADRα2-like* receptors) were identified [45,87]. However, only *Ci-ADRα2-a* was reported in neural structures, mainly in *Ci-TH* expressing cells, glutamatergic photoreceptors, and rostral trunk epidermal neurons, as well as GABAergic neurons located in the posterior sensory vesicle [45]. Most of these cell populations were investigated in the present work: WAY-100635 did not alter *Ci-TH* expression as well as photoreceptor differentiation as shown by *Ci-Opsin* results. Moreover, *Ci-Pans*, a neural marker particularly abundant in the posterior sensory vesicle where GABAergic neurons are present, was not affected by drug treatment. Finally, by using *Ci-Pou IV* as a marker for the peripheral nervous system, we observed that the rostral trunk epidermal neurons were the only epidermal sensory neurons well developed after WAY-100635 treatment. Thus, *Ci-ADRα2-a* expression did not reflect any of the observed effects induced by WAY-100635 exposure, suggesting that the malformations recorded in *C. intestinalis* were indeed determined by drug interaction with one or more types of 5HTRs.

Overall, our results provide new insight into 5HTR presence and distribution in an invertebrate model organism with a key phylogenetic position [88]. Moreover, by a pharmacological approach, we started to unravel the multifaceted roles of this amine in the development of the nervous system and the regulation of melanization of pigmented sensory organs. These data contribute to our better understanding of 5-HT pleiotropic roles in animal evolution, revealing an unexpected involvement in the formation of ascidian sensory cells.

## Data Availability

The data presented in this study are available on request from the corresponding authors.

## Supplementary Materials

The following supporting information can be downloaded at: https://www.mdpi.com/article/10.3390/cells12081150/s1, Additional File S1 Table S1: Sequence information of *Ciona* 5-HT receptors; Table S2: List of in situ hybridization probes and their synthesis information; Table S3: List of sequences used for reconstructing phylogenetic tree; Table S4: Expression levels (FPKM/RPKM) of *Ciona intestinalis* genes; Figure S1: Graphs showing the expression levels of Ciona intestinalis genes in different developmental stages; Additional File S2: FASTA file of the alignment used to generate the tree presented in Figure 1.
