# Supplementary material for: Serotonin Receptors and Their Involvement in Melanization of Sensory Cells in Ciona intestinalis
Source: Cells. 2023 Apr 13;12(8):1150. doi: 10.3390/cells12081150 (PMC10136630; doi:10.3390/cells12081150)
Supplement: Supplementary file 1 [file cells-12-01150-s001.zip › cells-2298577 - SM - Final/cells-2298577-Supplementary Material - additional file 2 .pdf]

>5HT4Rattusnorvegicus
VVLLTFFAMVILMAILGNLLVMVAVCRDRQLRKITNYFIVSLAFADLLVSVLVMPFGAIELVQDIW
FYGEMFCLVRTSLDVLLTTASIFHLCCISLDRYYAICCQPLVYRNKMTPLRIALMLGGCWVIPMFIS
FLPIMQGWNFCVFMVNKPYAITCSVVAFYIPFLLMVLAY-RIYVTAKEHAQQIQMLQRAE--
SRPQTADKTLCVIMGCFCFCWAPFFVTNIVDPFIDVPEKVWTAFLWLGYINSGLN
>5HT4Homosapiens
VVLLTFLSTVILMAILGNLLVMVAVCWDRQLRKITNYFIVSLAFADLLVSVLVMPFGAIELVQDIW
IYGEVFCLVRTSLDVLLTTASIFHLCCISLDRYYAICCQPLVYRNKMTPLRIALMLGGCWVIPTFISF
LPIMQGWNYCVFMVNKPYAITCSVVAFYIPFLLMVLAYYRIYVTAKEHAHQIQMLQRAE--
SRPQSADKTLCIIMGCFCLCWAPFFVTNIVDPFIDVPGQVWTAFLWLGYINSGLN
>5HT4Xenopustropicalis
IVLISFISAVILMTILGNLLVMVAVCRDRQLRKITNYFIVSLAFADLLVSVLVMPFGAIELVQEKWIY
GEMFCLVRTSLDVLLTTASILHLCCISLDRYYAICCQPLVYRNKMTPLRITLMLSGCWIIPTFISFLPI
MQGWNNCIFMVNKPYAITCSVVAFYIPFFLMVLAYYRIYITAREHARQIGVLQRAD--
HRHQHPDKTLCIIMGCFCLCWAPFFITNVVDPFINVPVELWTAFLWLGYINSGLN
>5HT2C1Cionarobusta
DWSVLLLIPLILMVATGNVLVIISVWLDRRLRSSTNYFLTSLAVADLLVAVVVMPPSLAMIVNNY
WPFPPQLCGVWTMLDVFFSTASILHLCLISLDRYVALSRP-
FSHRRSESTRSIGIRIFIVWATAFVIAVPLPILGASMCAIN-
VPEFAVFGSLVAFLLPLVIMFVMYTLTILALRRQAKLITNAMTQFG-VVIQ-
PDQVLGLIFVLFCLFWSPFFITNVVSHLCQLMGQCMNWFVWVGYVSSGVN
>5HT2C4Cionarobusta
DWSVLLLIPLILMVATGNVLVIISVWLDRRLRSSTNYFLTSLAVADLLVAVVVMPPSLAMIVNNY
WPFPPQLCGVWTMLDVFFSTASILHLCLISLDRYVALSRP-
FSHRRSESTRSIGIRIFIVWATAFVIAVPLPILGASMCAIN-
VPEFAVFGSLVAFLLPLVIMFVMYTLTILALRRQAKLITNAMTQFG-VVIQ-
PDQVLGLIFVLFCLFWSPFFITNVVSHLCQLMGQCMNWFVWVGYVSSGVN
>5HT2C5Cionarobusta
DWSVLLLIPLILMVATGNVLVIISVWLDRRLRSSTNYFLTSLAVADLLVAVVVMPPSLAMIVNNY
WPFPPQLCGVWTMLDVFFSTASILHLCLISLDRYVALSRP-
FSHRRSESTRSIGIRIFIVWATAFVIAVPLPILGASMCAIN-
VPEFAVFGSLVAFLLPLVIMFVMYTLTILALRRQAKLITNAMTQFG-VVIQ-
PDKVLGLIFVLFCLFWSPFFITNVVSHLCQLMGQCMNWFVWVGYVSSGVN
>5HT2C2Cionarobusta
DWSVLLLIPLILMVATGNVLVIISVWLDRRLRSSTNYFLTSLAVADLLVAVVVMPPSLAMIVNNY
WPFPPQLCGVWTMLDVFFSTASILHLCLISLDRYVALSRP-
FSHRRSESTRSIGIRIFIVWATAFVIAVPLPILGASMCAIN-
VPEFAVFGSLVAFLLPLVIMFVMYTLTILALRRQAKLITNAMTQFG-VVIQ-
PDKVLGLIFVLFCLFWSPFFITNVVSHLCQLMGQCMNWFVWVGYVSSGVN
>Ci5HT2C3Cionarobusta
DWSVLLLIPLILMVATGNVLVIISVWLDRRLRSSTNYFLTSLAVADLLVAVVVMPPSLAMIVNNY
WPFPPQLCGVWTMLDVFFSTASILHLCLISLDRYVALSRP-
FSHRRSESTRSIGIRIFIVWATAFVIAVPLPILGASMCAIN-
VPEFAVFGSLVAFLLPLVIMFVMYTLTILALRRQAKLITNAMTQFG-VVIQ-
PDQVLGLIFVLFCLFWSPFFITNVVSHLCQLMGQCMNWFVWVGYVSSGVN
>5HT2ARnorvegicus
NWSALLTTVVIILTIAGNILVIMAVSLEKKLQNATNYFLMSLAIADMLLGFLVMPVSMLTILYGYW
PLPSKLCAIWIYLDVLFSTASIMHLCAISLDRYVAIQNP-
IHHSRFNSRTKAFLKIIAVWTISVGISMPIPVFGLQSCLLA-
DDNFVLIGSFVAFFIPLTIMVITYFLTIKSLQKEATLCVS--DLSTQSSLS-
SEKVLGIVFFLFVVMWCPFFITNIMAVICKVIGALLNVFVWIGYLSSAVN
>5HT2AHsapiens

NWPALSIVIIIIMTIGGNILVIMAVSMEKKLHNATNYFLMSLAIADMLVGLLVMPLSLLAILYDYW
PLPRYLCPVWISLDVLFSTASIMHLCAISLDRYVAIRNP-
IEHSRFNSRTKAIMKIAIVWAISIGVSVPIPVIGLRTCVLN-
DPNFVLIGSFVAFFIPLTIMVITYCLTIYVLRRQALMLLHGHTECKRNTAE-
EEKVLGIVFFVFLIMWCPFFITNILSVLCELMEKLLNVFVWIGYVCSGIN
>5HT2BHsapiens
HWAALLILMVIIPTIGGNTLVILAVSLEKKLQYATNYFLMSLAVADLLVGLFVMPIALLTIMFEAW
PLPLVLCPAWLFLDVLFSTASIMHLCAISVDRYIAIKKP-
IQANQYNSRATAFIKITVVWLISIGIAIPVPIKGIETCVLTKEGDFMLFGSLAAFFTPLAIMIVTYFLTI
HALQKKAYLVKNKPPQDETPCSS-
PEKVLGIVFFLFLLMWCPFFITNITLVLCDTLQMLLEIFVWIGYVSSGVN
>5HT2BRnorvegicus
HWAALLIFAVIIPTIGGNILVILAVSLEKRLQYATNYFLMSLAVADLLVGLFVMPIALLTIMFEAWP
LPLALCPAWLFLDVLFSTASIMHLCAISLDRYIAIKKP-
IQANQCNSRTTAFVKITVVWLISIGIAIPVPIKGIETCELTKDGSFMLFGSLAAFFAPLTIMIVTYFLTI
HALRKKAYLVRNRPPQEDSSFSS-
PEKVLGIVFLFFLLMWCPFFITNVTLALCDTLKTLLQIFVWVGYVSSGVN
>5HT6Homosapiens
GWVAAALCVVIALTAAANSLLIALICTQPALRNTSNFFLVSLFTSDLMVGLVVMPPAMLNALYG
RWVLARGLCLLWTAFDVMCCSASILNLCLISLDRYLLILSP-
LRYKLRMTPLRALALVLGAWSLAALASFLPLLLGWHQCRLLASLPFVLVASGLTFFLPSGAICFTY
CRILLAARKQAVQVASLTTGL--
QVPRTPRLTLGILLGMFFVTWLPFFVANIVQAVCDISPGLFDVLTWLGYCNSTMN
>5HT6Rattusnorvegicus
GWVAAALCVVIVLTAAANSLLIVLICTQPALRNTSNFFLVSLFTSDLMVGLVVMPPAMLNALYG
RWVLARGLCLLWTAFDVMCCSASILNLCLISLDRYLLILSP-
LRYKLRMTAPRALALILGAWSLAALASFLPLLLGWHQCRLLASLPFVLVASGVTFFLPSGAICFTY
CRILLAARKQAVQVASLTTGL--
QVPRTPRLTLGILLGMFFVTWLPFFVANIAQAVCDISPGLFDVLTWLGYCNSTMN
>5HT6Gallusgallus
SWVAAFLCFIILLTTAGNFLLILLIVTQRSLRNTSNYFLVSLFMSDLMVGLVVMPPAMLNQLYGH
WVLRGDFCSLWYAFDVMCCSASILNLCVISLDRYLLIISP-
LKYKLRMTSCRALWLILATWTLAALASFLPIKLGWHQCRLVVSLPYALVASCLTFFLPSAAISFTY
CRILLAARKQAVQVASLASNE--
QVPHAPSLTLGILLGMFFVAWLPFFVTNVTQAVCDVPAGFFDVLTWLGYCNSTMN
>5HT1BMusmusculus
VLLVALLALITLATTLSNAFVIATVYRTRKLHTPANYLIASLAVTDLLVSILVMPISTMYTVTGRWT
LGQVVCDFWLSSDITCCTASIMHLCVIALDRYWAITDA-
VEYSAKRTPKRAAIMIVLVWVFSISISLPP-FFWR-
DCFVNTDVLYTVYSTVGAFYLPTLLLIALYGRIYVEARSRILKQTPNKTGT--
SSVTSINKTLGIILGAFIVCWLPFFIISLVMPICKFHMAIFDFFNWLGYLNSLIN
>5HT1BRattusnorvegicus
VLLVALLALITLATTLSNAFVIATVYRTRKLHTPANYLIASLAVTDLLVSILVMPISTMYTVTGRWT
LGQVVCDFWLSSDITCCTASIMHLCVIALDRYWAITDA-
VDYSAKRTPKRAAIMIVLVWVFSISISLPP-FFWR-
DCFVNTDVLYTVYSTVGAFYLPTLLLIALYGRIYVEARSRILKQTPNKTGT--
SSVTSINKTLGIILGAFIVCWLPFFIISLVMPICKFHMAIFDFFNWLGYLNSLIN
>5HT1BHomosapiens
VLLVMLLALITLATTLSNAFVIATVYRTRKLHTPANYLIASLAVTDLLVSILVMPISTMYTVTGRWT
LGQVVCDFWLSSDITCCTASILHLCVIALDRYWAITDA-
VEYSAKRTPKRAAVMIALVWVFSISISLPP-FFWR-

ECVVNTDILYTVYSTVGAFYFPTLLLIALYGRIYVEARSRILKQTPNRTGT--
SSVTSINKTLGIILGAFIVCWLPFFIISLVMPICKFHLAIFDFFTWLGYLNSLIN
>5HT1BGallusgallus
IVLAVVLALVTLATVLSNAFVIATVYQTRKLHTPANYLIASLAFTDLLVSILVMPISTLYTVTGKWT
LGQVVCDIWLSSDITCCTASILHLCVIALDRYWAITDA-
VEYSTKRTPKRAAGMIALVWVFSICISMPP-LFWR-
NCAVNTDVLYTVYSTVGAFYFPTLLLIALYGRIYVEARSRILKQTPKKAGS--
SSVTSINKTLGIILGAFIVCWLPFFIISLVLPICKFHMAIFDFFTWLGYLNSLIN
>5HT1DHomosapiens
ISLAVVLSVITLATVLSNAFVLTTILLTRKLHTPANYLIGSLATTDLLVSILVMPISIAYTITHTWNFG
QILCDIWLSSDITCCTASILHLCVIALDRYWAITDA-LEYSKRRTAGHAATMIAIVWAISICISIPP-
LFWR-DCLVNTSISYTIYSTCGAFYIPSVLLIILYGRIYRAARNRILNP-PSLYG---
SSLCSLNKILGIILGAFIICWLPFFVVSLVLPICRIHPALFDFFTWLGYLNSLIN
>5HT1DRattusnorvegicus
ISLVVVLSIITLATVLSNAFVLTTILLTKKLHTPANYLIGSLATTDLLVSILVMPISIAYTTTRTWNFGQ
ILCDIWVSSDITCCTASILHLCVIALDRYWAITDA-LEYSKRRTAGHAAAMIAAVWAISICISIPP-
LFWR-DCLVNTSISYTIYSTCGAFYIPSILLIILYGRIYVAARSRILNP-PSLYG---
SSLCSLNKTLGIILGAFIICWLPFFVVSLVLPICRIHPALFDFFTWLGYLNSLIN
>5HT1EHomosapiens
MLICMTLVVITTLTTLLNLAVIMAIGTTKKLHQPANYLICSLAVTDLLVAVLVMPLSIIYIVMDRW
KLGYFLCEVWLSVDMTCCTCSILHLCVIALDRYWAITNA-
IEYARKRTAKRAALMILTVWTISIFISMPP-
LFWRSQCTIQHDVIYTIYSTLGAFYIPLTLILILYYRIYHAAKSLYQKRGSSRHLKLTQTFCVSDRILG
LILGAFILSWLPFFIKELIVGLSIVSSEVADFLTWLGYVNSLIN
>5HT1EXenopustropicalis
MLISLTLAIITILTTVLNSAVILAICTTKKLHQPANYLICSLAVTDFLVAILVMPLSITYIVMDTWTLG
YVICEIWLSVDMTCCTCSILHLCVIALDRYWAITDA-IEYARKRTVKRAGIMILTVWTISVFISIPP-
LFWRNKCIIQHDLIYTIYSTCGAFYIPLTLILILYYRIYHAAKNLYQKRGSSRHLKLTQTFCVSERILG
LILGAFILSWLPFFIKELVVGIQLVSPEVADFLTWLGYVNSLVN
>5HT1FRattusnorvegicus
ILVSLTLSGLALMTTTINSLVITAIIVTRKLHHPANYLICSLAVTDFLVAVLVMPFSIVYIVSESWIMG
QGLCDLWLSVDIICCSCSILHLSAIALDRYRAITDA-VEYARKRTPRHAGITITTVWVISVFISMPP-
LFWR-QCIIKHDIVSTIYSTFGAFYIPLVLILILYYKIYRAARTLYHKRQAS-
RMKLVSTSYMLETTLGLILGAFVICWLPFFVKELVVNICEISEEMSNFLAWLGYLNSLIN
>5HT1FHomosapiens
ILVSLTLSGLALMTTTINSLVIAAIIVTRKLHHPANYLICSLAVTDFLVAVLVMPFSIVYIVRESWIM
GQVVCDIWLSVDITCCTCSILHLSAIALDRYRAITDA-VEYARKRTPKHAGIMITIVWIISVFISMPP-
LFWR-ECIIKHDIVSTIYSTFGAFYIPLALILILYYKIYRAAKTLYHKRQAS-
RIKSVSTSYVLETTLGLILGAFVICWLPFFVKELVVNVCDISEEMSNFLAWLGYLNSLIN
>5HT1FXenopustropicalis
ILISLTLSILTLMTTAINSLVIAAIIVTRKLHHPANYLICSLAVTDFLVAVLVMPFSIMYIMKETWIMG
QAICDIWLSVDITCCTCSILHLSAIALDRYRAITDA-VEYARKRTPKHAAFMIAVVWIISIFISMPP-
LFWR-ECIIKHDIVFTIYSTFGAFYIPLALILILYYKIYKAAKTLYHKRSVS-RVT--
TTLCIAETTLGLILGAFVICWLPFFVKEVIVNICEISDDMSNFLTWLGYLNSLIN
>5HT1AMusmusculus
VITSLLLGTLIFCAVLGNACVVAAIALERSLQNVANYLIGSLAVTDLMVSVLVLPMAALYQVLNK
WTLGQVTCDLFIALDVLCCTSSILHLCAIALDRYWAITDP-
IDYVNKRTPRRAAALISLTWLIGFLISIPPMLGWRAECTISKDHGYTIYSTFGAFYIPLLLMLVLYGRI
-
RAARFRIRKTVKKVEKNGQPGSGDCRKTLGIIMGTFILCWLPFFIVALVLPFCEMPELLGAIINWLG
YSNSLLN
>5HT1ARattusnorvegicus

VITSLLLGTLIFCAVLGNACVVAAIALERSLQNVANYLIGSLAVTDLMVSVLVLPMAALYQVLNK
WTLGQVTCDLFIALDVLCCTSSILHLCAIALDRYWAITDP-
IDYVNKRTPRRAAALISLTWLIGFLISIPPMLGWRTACTISKDHGYTIYSTFGAFYIPLLLMLVLYGRI
FRAARFRIRKTVRKVEKNGQPGSGDWRKTLGIIMGTFILCWLPFFIVALVLPFCEMPALLGAIINW
LGYSNSLLN
>5HT1AHomosapiens
VITSLLLGTLIFCAVLGNACVVAAIALERSLQNVANYLIGSLAVTDLMVSVLVLPMAALYQVLNK
WTLGQVTCDLFIALDVLCCTSSILHLCAIALDRYWAITDP-
IDYVNKRTPRRAAALISLTWLIGFLISIPPMLGWRTACTISKDHGYTIYSTFGAFYIPLLLMLVLYGRI
FRAARFRIRKTVKKVEKNGESGSRNWRKTLGIIMGTFILCWLPFFIVALVLPFCEMPTLLGAIINWL
GYSNSLLN
>5HT1AGallusgallus
LLTSLLLGTLILCAVSGNACVIAAIALERSLQTVANYLIGSLAVTDLMVSVLVLPMAALYQVLNK
WTLGQVICDIFISLDVLCCTSSILHLCAIALDRYWAITDP-
IDYVNKRTPRRAAVLISLTWLIGFLISIPPMLGWRTACTISKDHGYTIYSTFGAFYIPLLLMLVLYGRI
FKAARFRIRKTVKKAEKNGEPGKG-
WRKTLGIIMGTFILCWLPFFIVALVLPFCDMPEWLGAVINWLGYSNSLLN
>5HT1ADaniorerio
IIGSLFLAALILFAILGNACVIAAIALERSLQNVANYLIGSLAVTDLMVSVLVLPMAALYQVLNKW
TLGQEMCDIFISLDVLCCTSSILHLCAIALDRYWAITDP-
IDYVNKRTPRRAAILISLTWLIGFSISIPPMLGWRKACTISQDHGYTIYSTFGAFYIPLILMLVLYGRIF
RAARFRIRKTVKKTEKNGEVGKT-
WRKTLGIIMGTFIFCWLPFFIVALVLPFCQMPEWLGAVINWLGYSNSLLN
>5HT1AOncorhynchusmossambicus
VVTSFLLGALILCAIFGNACVVAAIALERSLQNVANYLIGSLAVTDLMVSVLVLPMAALYQVLNR
WTLGQIPCDIFISLDVLCCTSSILHLCAIALDRYWAITEP-
IDYMKKRTPRRAAVLISVTWLVGFSISVPPMLIMRSQCKIRQDPWYTIYSTFGAFYIPLTLMLVLYG
RIFKAARFRIRRTVRKTEKQGDTQAKSWKKTLGIIMGTFILCWLPFFIVALVMPFCQMPRWLEDVI
NWLGYSNSLLN
>5HT1AXenopuslaevis
IIASLFLGRSFSAGIFGNACVIAAIALERSLQNVANYLIGSLAVTDLMVSVLVLPMAAQNQVLNK
WTLGQVTCDIFISLDVLCCTSSILHLCAIALDRYWAITDP-
IDYVNKRTPRRAAVLISITWIVGFSISIPPMLGWRTACRISEDPGYTIYSTFGAFYIPLILMLVLYGKIF
KAARFRIRKTVKKAEKGAQQELEEVGKTLGIIMGTFILCWLPFFIVALVLPFCEMPHLLFDIITWLG
YSNSLLN
>5HT1.1Cionarobusta
IALAILLAIIIIGSMFGNILVVIAVRSERNLQTTANFLICSLAITDFLVACLVMPFSALYEISGTWVFG
DILCQTWTAIDIACCTASILHLCAIAFERHRSITSA-
VRYFSQGRRHTVAPKIVLVWVLAICISIPPILGWKNNVTTAAR-
EYTLYATLGSFYIPLALLLTAYVRIYVRIHQHIRRSESRTRLYWAKSARLWDKTLGTIVGAFVICWL
PFFAVTLAAAFCNMPHTLTSIVLWLGYCNSLVN
>5HT7Gallusgallus
IVIGAVLSIIILMTIAGNGLVIISVCIVKKLRQPSNYLVVSLAAADLSVAFAVMPFVTITDLVGGWLF
GKVFCNVFIAMDVMCCTASIMTLCIISVDRYLGITRP-
LTYPVRQNGKLMAKMVFIVWLLSASITLPP-
LFGWAVCLISQDFGYTVYSTGVAFYIPMAVMLVMYSRIYKAAKVSAEKHRFMNFS--
LEASSRGHRTLGIIVGAFTFCWFPFFLMSTARPFICLPLRLERTLLWLGYTNSLIN
>5HT7Xenopuslaevis
IVIGVVLSIITLFTIAGNALVIISVCIVKKLRQPSNYLVVSLAAADLSVAVAVMPFVIITDLVGGWLF
GKVFCNVFIAMDVMCCTASIMTLCVISVDRYLGITRP-
LTYPARQNGKLMAKMVFIVWLLSASITLPP-

LFGWAVCLISQDFGYTVYSTAVAFYIPMTVMLVMYQRIFVAAKISAEKHKFVNIP--
LEDKLPPKRTLGIIVGAFTFCWLPFFLLSTARPFICMPLRLERTLLWLGYTNSLIN
>5HT7Homosapiens
VVIGSILTLITLLTIAGNCLVVISVCFVKKLRQPSNYLIVSLALADLSVAVAVMPFVSVTDLIGGWIF
GHFFCNVFIAMDVMCCTASIMTLCVISIDRYLGITRP-
LTYPVRQNGKCMAKMILSVWLLSASITLPP-
LFGWAVCLISQDFGYTIYSTAVAFYIPMSVMLFMYYQIYKAARKSAAKHKFPGFP--LNG-----
TTLGIIVGAFTVCWLPFFLLSTARPFICIPLWVERTFLWLGYANSLIN
>5HT7Rattusnorvegicus
VVIGSILTLITLLTIAGNCLVVISVCFVKKLRQPSNYLIVSLALADLSVAVAVMPFVSVTDLIGGWIF
GHFFCNVFIAMDVMCCTASIMTLCVISIDRYLGITRP-
LTYPVRQNGKCMAKMILSVWLLSASITLPP-
LFGWAVCLISQDFGYTIYSTAVAFYIPMSVMLFMYYQIYKAARKSAAKHKFPGFP--LNG-----
TTLGIIVGAFTVCWLPFFLLSTARPFICIPLWVERTCLWLGYANSLIN
>5HT7Cionarobusta
ALIAALTSLLSLLIVVGNGLIIVSVALVKKLRQPANYLIVSLALSDFLVGLVVLPLTIVYDIMG-
WVFGPNVCDVHVSFDVICCTASIMNLCMISIDRYLMITQP-
MTYPKRRTGKLMLLLIATAWVLSCLVIIPA-
LFGFTACLISQERWFTIYSTLGAFYLPLAVMLCMYWKIYLEASRFNARHRLRSYSVVLNGHVIAKR
TLGIVVGAFTFCWLPFFIVTFLRPFACIPLWLVRFVLWLGYLNSALN
>5HT5AMusmusculus
VLVLTLLGFLAAATFTWNLLVLATILKVRTFHRVPHNLVASMAISDVLVAVLVMPLSLVHELS-
GWQLGRRLCQLWIACDVLCCTASIWNVTAIALDRYWSITRH-
LEYTLRTRKRVSNVMILLTWALSTVISLAPLLFGWGECQVSREPSYTVFSTVGAFYLPLCVVLFVY
WKIYRAAKFRMGSRKTNSVSNATQH---
PQLMVGILIGVFVLCWFPFFVTELISPLCSVPAIWKSIFLWLGYSNSFFN
>5HT5ARattusnorvegicus
VLVLTLLGFLAAATFTWNLLVLATILRVRTFHRVPHNLVASMAISDVLVAVLVMPLSLVHELS-
GWQLGRRLCQLWIACDVLCCTASIWNVTAIALDRYWSITRH-
LEYTLRARKRVSNVMILLTWALSAVISLAPLLFGWGECQVSREPSYTVFSTVGAFYLPLCVVLFVY
WKIYKAAKFRMGSRKTNSVSDASQH---
PQLMVGILIGVFVLCWFPFFVTELISPLCSIPALWKSIFLWLGYSNSFFN
>5HT5AHomosapiens
VLILTLLGFLVAATFAWNLLVLATILRVRTFHRVPHNLVASMAVSDVLVAALVMPLSLVHELS-
GWQLGRRLCQLWIACDVLCCTASIWNVTAIALDRYWSITRH-
MEYTLRTRKCVSNVMIALTWALSAVISLAPLLFGWGECQVSREPSYAVFSTVGAFYLPLCVVLFVY
WKIYKAAKFRVGSRKTNSVSDSAKQ---
PQLMVGILIGVFVLCWIPFFLTELISPLCSIPAIWKSIFLWLGYSNSFFN
>5HT5BMusmusculus
VLVVTLLVLLIVATFLWNLLVLVTILRVRAFHRVPHNLVASTAVSDVLVAALVMPLSLVSELSVG
WQLGRSLCHVWISFDVLCCTASIWNVAAIALDRYWTITR--
LQYTLRTRSRASALMIAITWALSALIALAPLLFGWGRCQVSQEPSYAVFSTCGAFYLPLAVVLFVY
WKIYKAAKFRFGRRRR-AVVEAPPE---
SEMMVGILIGVFVLCWIPFFLTELISPLCALPPIWKSIFLWLGYSNSFFN
>5HT5BRattusnorvegicus
VLVVTLLVLLIAATFLWNLLVLVTILRVRAFHRVPHNLVASTAVSDVLVAALVMPLSLVSELSAG
WQLGRSLCHVWISFDVLCCTASIWNVAAIALDRYWTITRH-
LQYTLRTRRRASALMIAITWALSALIALAPLLFGWGRCQVSQEPSYAVFSTCGAFYVPLAVVLFVY
WKIYKAAKFRFGRRRR-AVVEAPQE---
SEMMVGILIGVFVLCWIPFFLTELVSPLCALPPIWKSIFLWLGYSNSFFN
>5HT1.2Cionarobusta

ATVGFVFGVIILVGLVGNAVIITVIKREHKLHTRANYLIGSLAMADLLVSLLVSPFSAVMTLHEFFV
GHHVMCQLFTFLDVTCCTASILHLCAIAHDRYTAVT-K-
LQYRHRTHFKKVLPCIVLIWLAAVLLSVTPYFVFPSMCSVNTNKIYRIVATTIAFYAPLVIIIAIYWR
VARIAWTRIHHSVSATPSKAIRLCCHNDRTMGMVIGAFVACWLPFFIKELIVPFCGLDPSLEVFIN
WLGYANSALN
>5HTlikeCionarobusta
QVEIVFLLIVIVIGSIGNLLIIGSIRCERRLKNSGVGFITNLALADLAITAWYMPVVLANVLSGYVFE
GSWLCEFTGFLSCLCCEASLCTLMFISMDRYWKLIRP-
GSYETWFSKRSTLGWIAFIWIASFLIALPLIVGWQGSCMWNDEYGYNIFLVSTAIFVPLCATGFFYF
NIFAHVRRTRKKNATREIAS---
SIRAIQVVQNVRPHHSRHSSINSHKNARSKRVGEMLTLMVVVVFFVLFWCPIWR
>NP_058707.2metabotropicglutamatereceptor1Rattus norvegicus
MTVRGLLSAMRRLGVVGEFSLIGSDGWADKLQSPEENYVQDSKMGFVINAIYAMAHGLQNMH
HALCPGHVGLCDAMKPIDYDYVHVGTWHEGVLNIDDYKIQMNK-
EPCLKGQIKVIRKGEVSCCWICTACKEIPVRYLEWSDIESIIAIAFSCLGILVTLFVTLIFVLYRDTPVV
KSSSRQRLLVGLSSAMCYSALVTKTNRIARILAGRFMSAWAQVIIASILITLVVTLIIMEPPMPILSYP
SIKEV
