# Supplementary material for: Serotonin Receptors and Their Involvement in Melanization of Sensory Cells in Ciona intestinalis
Source: Cells. 2023 Apr 13;12(8):1150. doi: 10.3390/cells12081150 (PMC10136630; doi:10.3390/cells12081150)
Supplement: Supplementary file 1 [file cells-12-01150-s001.zip › cells-2298577 - SM - Final/cells-2298577-Supplementary Material, Additional_File1_done.pdf]

cells MDPI

Supplementary Material

Serotonin Receptors and Their Involvement in Melanization of Sensory Cells in *Ciona intestinalis*

Silvia Mercurio [1], Matteo Bozzo [2], Alessandro Pennati [3], Simona Candiani [2,*] and Roberta Pennati [1,*]

[1] Department of Environmental Science and Policy, Università degli Studi di Milano, 20133 Milan, Italy; sil.mercurio@gmail.com
[2] Dipartimento di Scienze della Terra, dell'Ambiente e della Vita, Università degli Studi di Genova, 16132 Genoa, Italy; matteo.bozzo@unige.it
[3] Institute of Zoology and Center of Molecular Biosciences, University of Innsbruck, 6020 Innsbruck, Austria; alessandro.pennati@uibk.ac.at
* Correspondence: simona.candiani@unige.it (S.C.); roberta.pennati@unimi.it (R.P.); Tel.: +39-0103358051 (S.C.); +39-0250314765 (R.P.)

Table S1. Sequence information of *Ciona* 5-HT receptors.

Name	Accession
Ciona intestinalis 5HT1.1	XP_018672564.2
Ciona intestinalis 5HT1.2	XP_018668983.1
Ciona intestinalis 5HT2 (five isoforms)	XP_026694550.1<br>XP_026694549.1<br>XP_026694547.1<br>XP_018671404.1<br>XP_018671402.1
Ciona intestinalis 5HT7	XP_002123484.1
Ciona intestinali 5HT-like	XM_002130995.3

Table S2. List of *in situ* hybridization probes and their synthesis information.

Gene/Transcript	Probe synthesis	Reference
Ci-Pans	*885bp probe amplified from cDNA corresponding to:*<br>clone: citb043k05	Alfano et al., 2007
Ci-Synapsin	*1680bp probe amplified from cDNA with primers:*<br>Fwd: CTAAAATAAAGATGTCTTCAATGC<br>Rv: ATATTCGCGCTATTGCAAGC	Candiani et al., 2010
Ci-POU IV	*1139bp probe amplified from cDNA corresponding to:*<br>clone: citb034g05	Candiani et al., 2005
Ci-TH	*1145bp probe amplified from cDNA with primers:*<br>Fwd: AGTAAGAGCGGATTTTGGAAGA<br>Rv: ACGTTTGGTGCAGTGTTGTG	Zega et al., 2010
Ci-Opsin1	*567bp probe amplified from cDNA with primers:*<br>Fwd: TGCTAACGGTGCGGGATATT<br>Rv: CCAAGCGCGTAATCGATCTG	Kusakabe et al., 2001
Ci-Six 3/6	*438bp probe amplified from cDNA with primers*:<br>Fwd: TCACCCAACCCTTTGCTACC<br>Rv: GGCTTCGGGTTCGTTCCTTA	Mazet et al., 2005
Ci-Tyr	*356bp probe amplified from cDNA with primers:*<br>Fwd: GACGAGTCAAAACGCGTCAC<br>Rv: GACTACGTCACGCAGCTCTT	Esposito et al., 2012
Ci-Tyrp 1/2	417bp *probe amplified from cDNA with primers*:<br>Fwd: GACGAACTTCTCGGAGGGCA<br>Rv: GATCGGATCATTTGCGGAGG	Esposito et al., 2012

Ci-Rab 32/38	456bp *probe amplified from cDNA with primers*:<br>Fwd: TGTGATCGGCGAGTTAGGTG<br>Rv: AGTTTCGAACCAAGCCACGA	Racioppi et al., 2019
Ci-Tcf	*probe amplified from Ciona Gene Collection Release 1:*<br>R1CiGC13p03	Squarzoni et al., 2011
Ci-5HT-like	*1403 bp probe amplified from cDNA with primers*:<br>Fwd: ACCGCATACATGGCAGTTG<br>Rv: ACGGCGGTAAAAAGTAAAA	
Ci-5HT1.1	1039bp *probe amplified from cDNA with primers*:<br>Fwd: TTTCTTGGTCGCGTGCCTCGTAAT<br>Rv: CCCTGTCGTCTGCGCTCGTCTT	
Ci-5HT1.2	*1447 bp probe amplified from cDNA with primers:*<br>Fwd: CTGTCAGTCACGCCTTATTTCG<br>Rv: ATCTTTATTATCGCCTTCTGTTCC	
Ci-5HT7	1333bp *probe amplified from cDNA with primers*:<br>Fwd: AAGGCGACGCTATGCTCACAACAG<br>Rv: TCGCCCGAAGAAGACACCGTATC	
*Ci-5HT2**	1040bp *probe amplified from cDNA with primers*:<br>Fwd: TATATGTTACAGAGGCGATGGATT<br>Rv: TTGGCAAAGATGGCTGACTAC	

*The primers used were designed to amplify all 5HT2 isoforms.

Table S3. List of sequences used for reconstructing phylogenetic tree.

**Name/Species**	**Accession number**
5HT1A *Danio rerio*	NP_001116793
5HT1A *Xenopus laevis*	NP_001079299
5HT1A *Gallus gallus*	NP_001163999
5HT1A *Oreochromis mossambicus*	AAP83427
5HT1A *Homo sapiens*	NP_000515
5HT1A *Mus musculus*	Q64264
5HT1A *Rattus norvegicus*	NP_036717
5HT1B *Gallus gallus*	ADC54223
5HT1B *Mus musculus*	NP_034612
5HT1B *Rattus norvegicus*	NP_071561
5HT1B *Homo sapiens*	NP_000854
5HT1D *Homo sapiens*	NP_000854
5HT1D *Rattus norvegicus*	NP_036984
5HT1E *Homo sapiens*	NP_000856
5HT1E *Xenopus tropicalis*	XP_002933964.1
5HT1F *Homo sapiens*	NP_000857.1
5HT1F *Rattus norvegicus*	NP_068629
5HT1F *Xenopus tropicalis*	XP_002931817.1
5HT1.1 *Ciona intestinalis*	XP_018672564.2
5HT1.2 Ciona intestinalis	XP_018668983.1
5HT2A *Rattus norvegicus*	NP_058950
5HT2B *Homo sapiens*	NP_000858
5HT2B *Rattus norvegicus*	NP_058946
5HT2A *Homo sapiens*	NP_000859
5HT2 *Ciona intestinalis*	XP_026694550.1<br>XP_026694549.1<br>XP_026694547.1<br>XP_018671404.1<br>XP_018671402.1
5HT4 *Xenopus tropicalis*	XP_002939852.1
5HT4 *Homo sapiens*	NP_000861.1

5HT4 *Rattus norvegicus*	NP_036985.2
5HT5A *Homo sapiens*	NP_076917
5HT5A *Rattus norvegicus*	NP_037280
5HT5A *Mus musculus*	NP_032340
5HT5B *Mus musculus*	NP_034613
5HT5B *Rattus norvegicus*	NP_077371
5HT6 *Homo sapiens*	NP_000862
5HT6 *Rattus norvegicus*	NP_077341
5HT6 *Gallus gallus*	NP_001166911
5HT7 *Gallus gallus*	NP_001165240
5HT7 *Xenopus laevis*	NP_001079253
5HT7 *Homo sapiens*	CAH69968
5HT7 *Rattus norvegicus*	NP_075227
5HT7 *Ciona intestinalis*	XP_002123484.1
metabotropic glutamate receptor 1 *Rattus norvegicus*	NP_058707.2

Table S4. Expression levels (FPKM/RPKM) of *Ciona intestinalis* genes as reported in Aniseed database (http://www.aniseed.cnrs.fr/, last accessed 27 March 2023).

**Replica 1: experiment n°34135**					
**Stage/Gene**	**5HTR1.1**	**5HTR1.2**	**5HTR2**	**5HTR7**	5HTR-like
Stage 0 (Unfertilized egg)	0.000	1.189	7.209	0.095	105.531
Stage 8 (64-cell)	0.092	0.788	7.432	0.161	88.116
Stage 11 (early gastrula)	0.648	0.361	4.743	0.125	83.815
Stage 12 (mid gastrula)	0.449	0.733	4.271	0.000	74.139
Stage 15 (mid neurula)	0.211	1.175	4.634	0.245	46.270
Stage 21 (mid tailbud I)	0.848	0.577	3.621	0.246	18.460
Stage 26 (hatching larva)	0.165	0.795	4.218	1.622	18.460
Replica 2: experiment n°34135					
**Stage/gene**	5HTR1.1	5HTR1.2	5HTR2	5HTR7	5HTR-like
Stage 0 (Unfertilized egg)	0.000	0.699	2.386	0.084	94.597
Stage 8 (64-cell)	0.350	0.293	3.848	0.152	79.170
Stage 11 (early gastrula)	0.532	0.461	3.243	0.257	89.889
Stage 12 (mid gastrula)	1.213	0.554	2.219	0.162	68.334
Stage 15 (mid neurula)	0.653	0.768	3.584	0.631	37.485
Stage 21 (mid tailbud I)	0.174	0.453	1.594	0.707	16.484
Stage 26 (hatching larva)	0.000	0.523	0.86	1.090	13.786

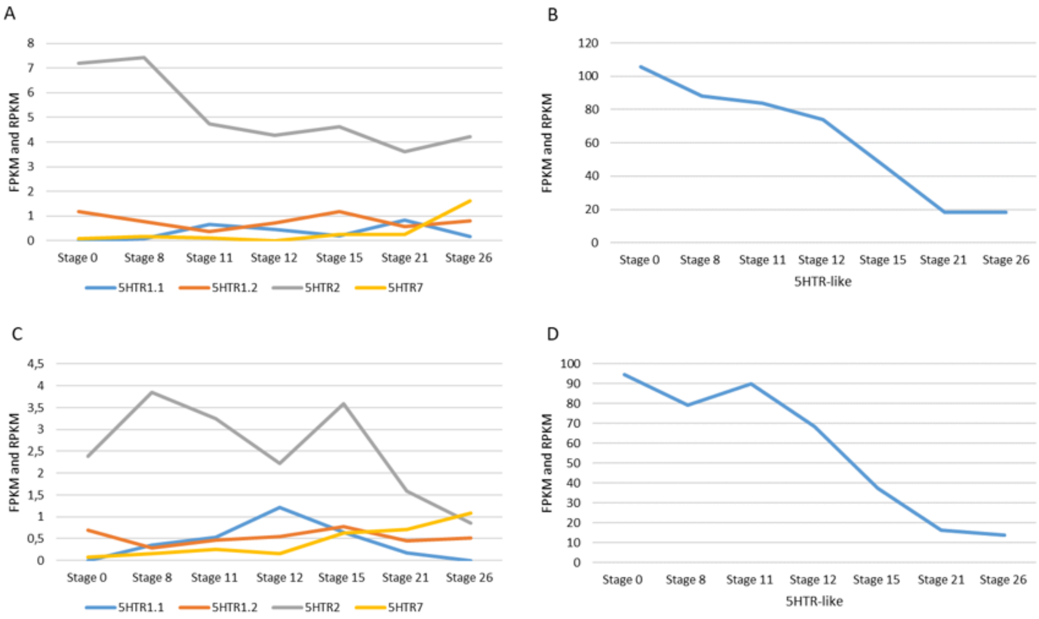

Figure S1. Graphs showing the expression levels of *Ciona intestinalis* genes in different developmental stages as reported in Aniseed database (http://www.aniseed.cnrs.fr/, last accessed 27 March 2023). A,B Replica 1, experiment n°34135; C,D Replica 2, experiment n°34135.
